# On the security of consumer wearable devices in the Internet of Things

**DOI:** 10.1371/journal.pone.0195487

**Published:** 2018-04-18

**Authors:** Hasan Tahir, Ruhma Tahir, Klaus McDonald-Maier

**Affiliations:** 1 School of Electrical Engineering and Computer Science (SEECS), National University of Sciences and Technology (NUST), Islamabad, Pakistan; 2 Embedded and Intelligent Systems Research Laboratory, School of Computer Science and Electronic Engineering, University of Essex, Wivenhoe Park, Colchester CO4 3SQ, United Kingdom; University of Texas at San Antonio, UNITED STATES

## Abstract

Miniaturization of computer hardware and the demand for network capable devices has resulted in the emergence of a new class of technology called wearable computing. Wearable devices have many purposes like lifestyle support, health monitoring, fitness monitoring, entertainment, industrial uses, and gaming. Wearable devices are hurriedly being marketed in an attempt to capture an emerging market. Owing to this, some devices do not adequately address the need for security. To enable virtualization and connectivity wearable devices sense and transmit data, therefore it is essential that the device, its data and the user are protected. In this paper the use of novel Integrated Circuit Metric (ICMetric) technology for the provision of security in wearable devices has been suggested. ICMetric technology uses the features of a device to generate an identification which is then used for the provision of cryptographic services. This paper explores how a device ICMetric can be generated by using the accelerometer and gyroscope sensor. Since wearable devices often operate in a group setting the work also focuses on generating a group identification which is then used to deliver services like authentication, confidentiality, secure admission and symmetric key generation. Experiment and simulation results prove that the scheme offers high levels of security without compromising on resource demands.

## 1 Introduction

Recently there has been a rapid increase in the number internet capable devices. A paradigm shift in computing has caused rapid development of personal devices. The latest trend in computing aims to interconnect sensors, devices, embedded systems, software and hardware to collect and exchange data. The Internet of Things (IoT) allows devices to connect to the internet thus creating a ubiquitous environment where devices share data and information for increased connectivity. IoT devices comprise an embedded system with valuable data which is being communicated often via short range wireless signals like Bluetooth. The IoT is a dense population of interconnected smart devices like smartphones, televisions, vehicles, health monitoring devices, home automation systems and wearable devices to name a few. Interconnectivity, varying purpose and capability creates a compelling case for ensuring security and safety of devices in the IoT to protect the users data and privacy.

Wearable computing is an area that is closely related to pervasive and ubiquitous computing. Consumer wearable devices are being marketed that can be worn on the body for both business and personal use. What sets the wearable technology apart from conventional mobile devices is the fact that these devices are designed to be worn on the body and not carried. Wearable devices are also designed to augment knowledge and learning by enhancing users experience. By design these are miniature electronic devices composed of multiple sensors controlled by a microcontroller. The sensors can for example sense movement, physiological signals, atmospheric changes like temperature and humidity. The most common sensors found in modern wearable devices are accelerometers and gyroscopes. Lifestyle, health monitoring, fitness monitoring, entertainment, gaming and industrial support wearable devices cannot provide the wide range of features without these sensors.

- Health monitoring—devices embedded with sensors which can detect changes in the physiological signals of the body. These devices can be worn permanently by the user.
- Fitness monitoring—devices intended to enhance sports and fitness related activities through monitoring and tracking. Unlike health monitoring these devices are not intended to be worn by the user at all times.
- Lifestyle—general purpose wearable articles like smart watches and smart clothing which can provide cellular, internet connectivity etc.
- Entertainment—wearable devices that can stream audio and video. The devices in this category can be wireless headphones, speakers, and wearable displays with the ability of connecting to wide range of entertainment systems like smart TV’s, digital media players, home theatre systems etc.
- Gaming—devices designed to enhance user experience during gameplay. Advanced devices are intended to create an immersive augmented environment through the use of 3D head wearable and walkable surround displays.
- Industrial support—devices that fall in this category are designed to aid the user in performing their task safely while increasing productivity and efficiency. These devices are intended to be worn by professionals in an industrial setting.

Consumers can purchase an internet capable version of almost every home appliance; but many devices still do not possess the ability nor the resources required for the provision of security. Devices that possess poor physical security, authentication, encryption, interfaces can become a target of spoofing, key theft and identity theft. Conventional security algorithms rely on stored cryptographic keys. If the keys are exposed then the system and its data is compromised. The ICMetric technology aims to resolve the issue of key theft by using the features of a device to create a device identification which can then be used for cryptographic key generation.

This paper studies the ICMetric technology as a key theft deterrent and as a basis for cryptographic services. The paper proposes a security framework that targets devices communicating in the multiparty environment. The research aims to make four essential contributions.

- The first contribution is a demonstration of how an accelerometer and gyroscope bias which can be used to generate a device ICMetric.
- The second contribution of the research is an authentication scheme that can be used to authenticate individual devices by using their ICMetric. This is a particular challenge as essential properties need to be preserved.
- The third contribution of the research is the creation of a group ICMetric that is computed by using a device ICMetric. The challenge is computing the group ICMetric without transmitting the individual device ICMetric.
- The fourth contribution of the research is a scheme that generates a symmetric key for the group. The scheme is based on prominent cryptographic primitives which facilitate in generating keys of various sizes. This implies that the scheme is adaptable to the varying needs and resources of a device.

The paper has been structured by first introducing wearable devices and related work that is happening in the field of IoT. The paper then introduces the ICMetric technology and shows how a device ICMetric can be generated using the accelerometer and gyroscope sensors. The paper presents a detailed study of the proposed scheme by providing the imprinting, authentication, group ICMetric generation and the key generation algorithm. The paper concludes with the simulation results and outcomes of the study.

## 2 Related work

A recent study on consumer devices in the IoT shows that devices are being sold with little or no security provision [1]. Those devices that do have security services have been poorly designed. This creates the compelling case that security for IoT devices is of utmost importance and needs a fresh approach to ensure the highest levels of security. Given below is a broad classification of wearable devices.

Recent research shows the capabilities of consumer wearable devices and also identifies barriers to their adoption [2]. The authors first explain the capabilities of wearable health devices and also bring to light three prominent issues related to these wearables i.e. safety, reliability and security. The authors highlight that a well-coordinated cyber-attack could lead to data being compromised, lost or distorted.

Wearable devices are being used and experimented in a variety of healthcare related application [3] [4] [5]. Although the applications are novel they lack security implementations which is a concern since the applications target healthcare applications of body wearables. In another research authors present a study on how wearable devices can improve working of challenging environments like hospital wards [6]. They present a case study in which they conclude that wearable devices would enhance the level of usability and context awareness. The authors have identified four security challenges facing wearable devices i.e. confidentiality, authentication, hostile environment and device network security. A recent research by Kumar et al [7] studies fall detection through inertial sensing. The authors design a wearable device embedded with an accelerometer, gyroscope and Bluetooth. Being a health monitoring sensor the system continuously senses motion related variables, but the system lacks any form of security implementation.

Authentication is a basic requirement for any security system. Researchers have developed a scheme which can detect the wearer of a wearable device by using a bioelectrical impedance signal [8]. The research shows that it is possible to use a wrist wearable health sensor called the Shimmer sensor to uniquely identify a user through their physiological signals [9]. The proposed scheme possesses a 98% successful authentication rate but the scheme does not offer other necessary security services like integrity and confidentiality. The provision of authenticity alone is a false promise of security and hence the work needs extension.

A recent research shows that even widely marketed wearable devices can possess poor security provisions which makes attacking them an effortless task [10]. The paper studies the Fitbit tracker that has 96KB RAM and is embedded with an accelerometer sensor, altimeter sensor. The paper studies the security of the Fitbit tracker and shows that it is possible to attack the wearable device by exploiting weaknesses in the system. The authors reverse engineer the Fitbit and observe that it lacks security provisions. For instance the tracker transmits user credentials in plain text. Besides this any HTTP data processing that takes place is also in plaintext. The authors also demonstrate that counterfeit data can be generated and injected into the tracker by attaching it to moving objects like the wheel of a car. Authenticating wearable devices using body and gait recognition is a concept which has been explored [11] [12]. Chauhan et al. [13] in their paper design a security scheme for the latest optical wearable device Google Glass. The authors present an unobtrusive security scheme that uses multiple user gestures to establish the authenticity of the user. Although the concept is interesting, it has a weakness that it requires user intervention for authenticity. The other weakness of the scheme is that the user should have prior experience with the device for improved accuracy.

Presently two forms of security implementations targeting security for wearable devices have been identified [14]. The first form of security attempts to authenticate the user wearing the device. The second form of security for wearable devices aims to identify the device and thus secure it. Research on the emerging ICMetric technology proves that it is both possible and recommended to use the device features for identifying a device [15]. The technology has been researched by coupling with a Shimmer sensor for the provision of security in a single user environment. Tests prove that the scheme can offer authentication, confidentiality and integrity in a resource constrained environment.

Cryptographic keys are a crucial part of a security algorithm which is why they are kept secret. If the keys are exposed then the security of the system can be compromised. A technology that has been experimented with as a novel root of trust is Physically Unclonable Functions (PUF). A PUF is a one way function that is based on a physical property and also holds the quality that it is unclonable [16]. When a PUF is provided an input it produces an unpredictable output that is based on a unique physical system characteristic. Thus a PUF is a challenge response function that is simple to evaluate but is difficult to predict. These qualities exist due to the fact that PUF’s are embodied in a physical structure that is beyond the control of even device manufacturers. If a PUF is given input (challenge) a standard stimulus, then each PUF instantiation will produce a unique response. Early research [17] on optical PUF showed that when a stationary scattering medium is placed in the path of a laser beam then a unique splatter pattern is created which is a repeatable device response. This research was the foundation of the PUF technology but had limited applications owing to design complexity. MEMS sensors are also suitable for PUF implementation. When a sensor is embedded onto the main chip it introduces variations(bias) that are beyond the control of even the manufacturer. This means that it is not possible to create two chips that behave the same even if the underlying technology and methods remain the same. Experiments on PUF have shown that the technology can be used for the provision of cryptographic services. This creates hardware entangled cryptography since the features used are often based on variability in chip manufacturing [18].

## 3 Integrated Circuit Metric (ICMetric)

In an age when computing power has become not only common but also cheap, cryptographers are trying to stay ahead of attackers by increasing key sizes. Often the purpose of increasing the key size is to make it computationally infeasible for an attacker to brute force the keys. Keys are often stored on the system, which makes them a target for attackers. Any effort to increase the key size as a way to protect the system is rendered useless if the system is successfully attacked and the keys are captured.

Traditional device fingerprinting techniques are designed to enable system identification but can also be used to identify users on the web. Broadly these can be placed in one of two models either client based or server based [19]. In client based method; the device features are extracted by installing a software on the target system. The problem with this method is that installing a software requires user permission which may not always be possible since many users and organizations prohibit software installations. Installing a software is also not favoured as it may conceal malware in a seemingly meaningful application.

When using the server based method a software installation is not required because device identifications are generated by gathering device characteristics which are readily available and do not require user permission. The problem with this technique is that the device identifications are assembled using relatively simpler features which do not ensure diversity and entropy. Features used in the server method can be extracted and reproduced by attackers thus aiding spoofing.

IoT devices are rooted in the physical world while communications happen over a network. A threat actor in the IoT could be an internal or external entity, while the attack could be intentional or accidental. When a device in the IoT is compromised then critical data can be captured, lives can be lost and disruptions can be caused as interconnected devices and systems fail.

The Integrated Circuit Metric (ICMetric) technology has been conceived as an alternative method to stored keys and as a basis for cryptographic services [20] [21]. The unique concept and design of the ICMetric technology does not limit its use as an alternative method to stored keys. The technology can also be used as a basis for key generation and for preventing impersonation and spoofing based attacks on computation systems. By using the ICMetric technology there is no need to store the keys or any associated templates because the ICMetric and keys are generated when required and discarded thereafter. Doing so discourages attackers since there is no cryptographic key present on the system.

Incorporating the ICMetric technology into group communications ensures that only trusted parties form part of the group setting. Thus any device that is not part of the established ICMetric group is considered a malicious entity.

### 3.1 ICMetric generation

**Fig 1:**
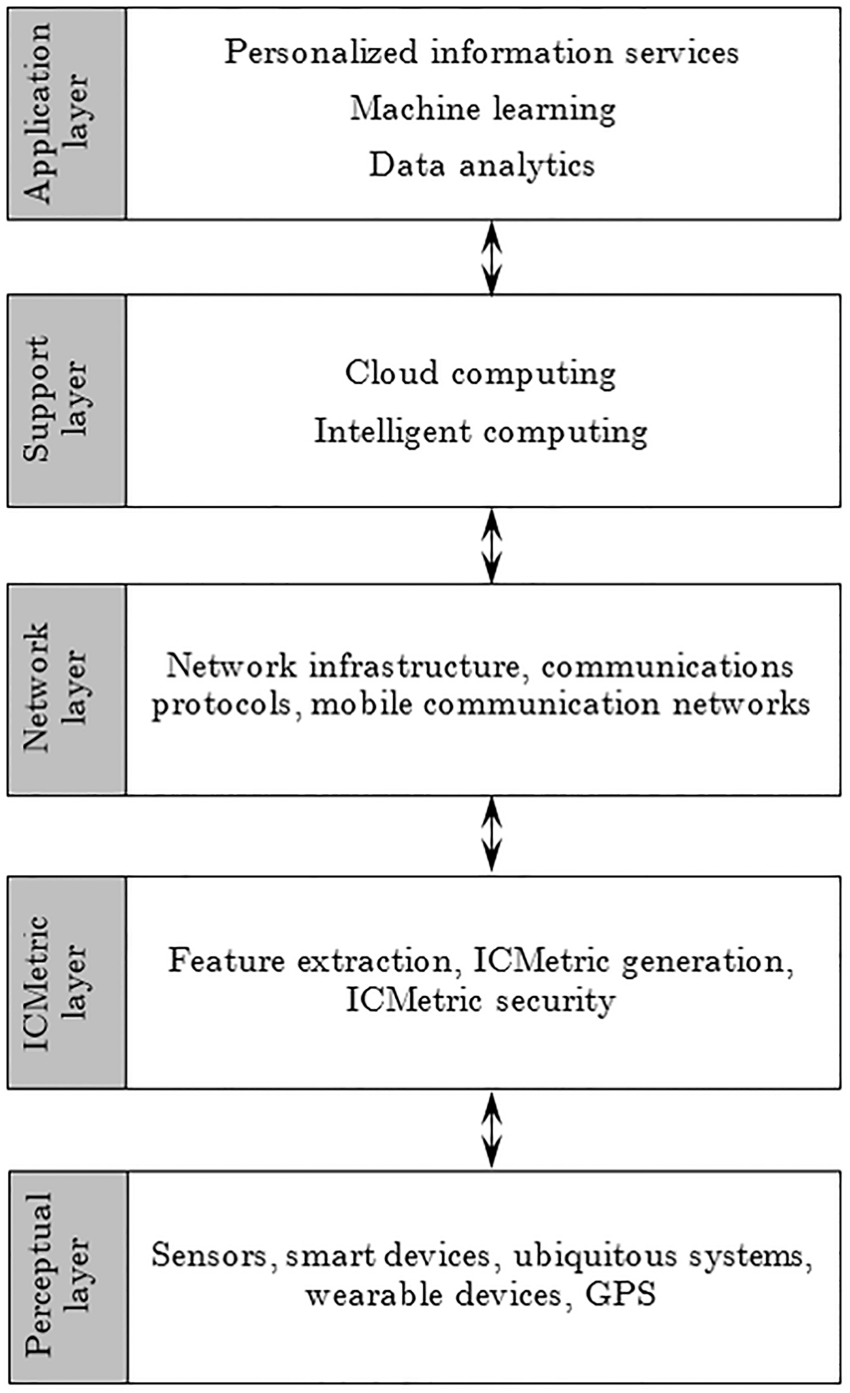
ICMetric based IoT architecture.

The ICMetric technology has been designed as an additional security layer in the existing communication architecture hence there is no need to rewrite communication architectures. ICMetric is a patented technology and research has shown that it can be used for key generation [22] [20]. The ICMetric layer conceals single and multiparty systems with a protective layer so that any communications to and from the system are based on ICMetric security. Fig 1 is an IoT architecture with the ICMetric layer. The proposed architecture is an adaptation of the IoT architecture proposed by Suo et al [23]. By using the ICMetric technology a device fingerprint (called an ICMetric) is created which is then used to generate the cryptographic keys of the system. The ICMetric of a device is generated by first measuring unique system features under a standard stimulus. The system features are then processed statistically to produce credentials that are combined to produce a final ICMetric. Provision of cryptographic services is then based on this established ICMetric.

Unlike conventional device fingerprints, the ICMetric is generated by using both hardware and software features. If the hardware or software environment of a device is maliciously tampered in anyway then the generated ICMetric is different from the original. The ICMetric of a device should possesses certain qualities which add to the complexity of the generation and validation process. The ICMetric must hold the following essential properties:

- The ICMetric must be generated when required and discarded thereafter.
- The ICMetric cannot be stored on a system.
- The ICMetric cannot be transmitted or communicated even to trusted parties.
- The ICMetric cannot be directly used as a cryptographic key.
- The ICMetric should be composed of device features that cannot be predicted or spoofed.

The ICMetric generation [22] [20] is a two phase process i.e. calibration phase and operation phase.

#### 3.1.1 MEMS based ICMetric generation

In this paper MEMS sensors have been studied for the creation of a device ICMetric. To generate an ICMetric for a device which is embedded with MEMS sensors a standard stimulus is provided as input. The sensor responds to the stimulus and provides an output that is reflective of the underlying bias in the sensor. Thus the sensor bias is a unique feature for the ICMetric generation. To establish the device ICMetric the obtained responses to a stimulus are subjected to statistical measures. The statistical measures include confidence interval, standard deviation, interquartile range and skewness. The statistical indicators create credentials which can be combined to generate a unique device ICMetric. The combination of individual credentials has been discussed in section 3.3 of the paper.

### 3.2 Calibration phase

In the calibration phase, feature values are read from the device under the specified stimulus. The values are tabulated and frequencies are computed based on the readings provided by the device. After this the unimodal distributions are generated for each feature. Statistical values are generated from the unimodal distribution. Thus the calibration phase is applied to extract the desired feature values and generate a unimodal distribution. The statistical values serve as credentials that form the device ICMetric. Thus the purpose of the calibration phase is to establish the basic feature values required for the creation of a final ICMetric through the operation phase.

### 3.3 Operation phase

The operation phase generates a final device ICMetric using the statistical values generated in the calibration phase. The purpose of the operation phase is to create a final device ICMetric by applying a function on the established statistical credentials. The ICMetric is generated by either using the feature addition technique or the feature concatenation technique. In the feature addition technique, the individual statistical values are added to generate an ICMetric which is diverse but small in size. The feature concatenation technique generates a device ICMetric by concatenating the individual statistical values. The resulting number lacks diversity but can be fairly large in size depending on the number of digits per statistical value.

### 3.4 Statistical analysis of device features

To generate the ICMetric those features are selected which can be represented as a unimodal distribution (a distribution with one clear peak). This is an essential requirement as it ensures that the target feature value is not erratic and lies within a deterministic range. The unimodal distribution is analyzed statistically so that credentials can be obtained for the operation phase. Statistical credentials are obtained by determining the mean, standard deviation, confidence interval, interquartile range and skewness. Each statistical measure is strongly dependent on the readings obtained from the sensors.

Statisticians often question where the mean would lie in a population based on a certain confidence level. This type of indication is of particular importance when a curve does not follow the normal distribution. The confidence interval determines the interval in which the population mean would lie based on a confidence level [24]. To generate the ICMetric we use the 95% confidence interval. If $\bar{X}$ is the mean, *σ* is the standard deviation and *n* is the total number of observations then the confidence interval *CI* is dependent on a two sided multiplier 1.96. The confidence interval is given by:

$$\begin{array}{c} 95\%\;CI=\bar{X}\pm 1.96\frac{\sigma }{\sqrt{n}} \end{array}$$ (1)

Each statistical measure gives important insight into the readings obtained from the sensor. For instance the mean of a population shows where an average reading would lie in the population. The standard deviation indicates how widely dispersed the readings are compared to the population mean. Hence a low standard deviation indicates that the data points are close to the mean.

## 4 Determining a sensor bias

### 4.1 MEMS sensors

Micro Electrical and Mechanical System (MEMS) is a technology that mimics conventional electrical and mechanical systems at a micro scale. There are many MEMS based sensors that are being embedded into modern smartphones, laptops, vehicles and wearable devices. The most widely used and best examples of MEMS sensors are the accelerometer and the gyroscope. Accelerometers are designed to measure the acceleration of an object, while gyroscopes measure angular velocity.

MEMS sensors possess a bias that affects their functioning. A sensor bias is defined as the difference between the test result and the expected result [25]. Hence the bias in a measuring instrument is the result of single or multiple systematic errors in the system. There are many bias inducing factors for example the fabrication process where slight stresses are introduced when a sensor is mounted onto the main board. Even the smallest imperfections in MEMS sensors has an impact on the accuracy and precision of the sensor. Calibrations attempt to compensate for the error in the readings by incorporating a linear value into the raw values obtained from the sensor but fail to fully eliminate the bias. Research has proven that it is both possible and recommended to use this sensor bias for identifying a device [26] [27] [28] [29] [30].

### 4.2 Accelerometer and gyroscope offset measurement

The ICMetric should be generated without user intervention and the identification should be repeatable. These requirements influence the choice of sensors and the choice of stimulus. A repeatable offset can only be produced if a device experiences the same stimulus for the entire duration of the bias identification process. To determine the accelerometer and gyroscope bias both devices must be put on a stable surface free from movement, rotation and vibration. Hence the datum point for the accelerometer and the gyroscope is 0 *m*/*sec*^2^ and 0 *deg*/*sec* respectively. To extract the accelerometer and gyroscope bias, a body wearable device is required which is equipped with the sensors. The Shimmer sensor is a Bluetooth ready, wearable health device which is equipped with a range of sensors like EMG, ECG, GSR, magnetometer, tri axial accelerometer and tri axial gyroscope.

**Fig 2:**
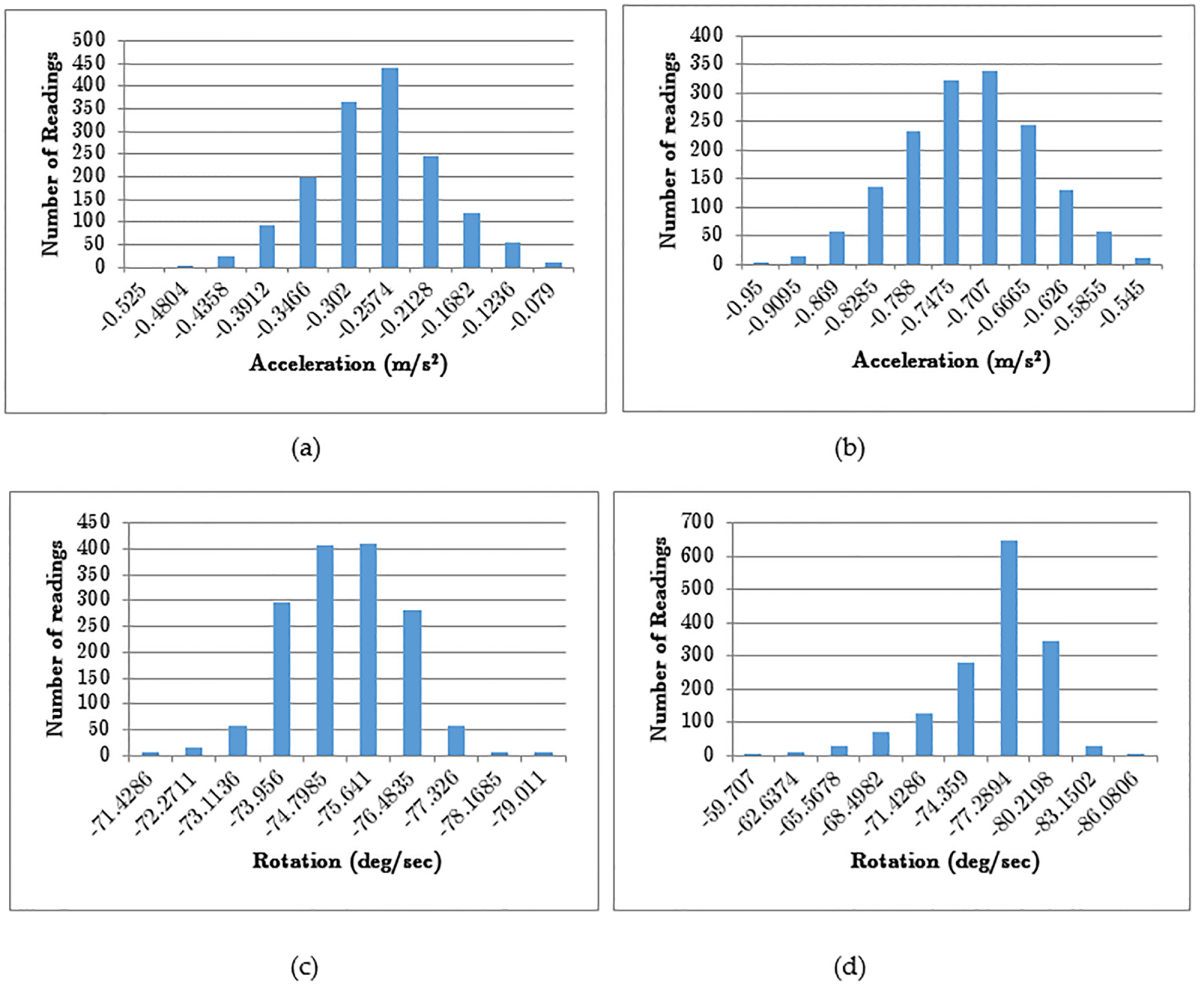
Calibrated unimodal distributions for two different devices evaluating x-axis (a) accelerometer (b) accelerometer (c) gyroscope (d) gyroscope.

The experimental setup consists of 5 identical shimmer sensors placed on a vibration free surface. The experiment aims to prove that a sufficient bias exists in each sensor and that the statistical characteristics vary between identical sensors. To generate a consistent ICMetric, 1500 individual axis readings were obtained from the accelerometer and gyroscope. The readings are obtained at regular intervals in a CSV file that holds both RAW and calibrated readings from the accelerometer and the gyroscope. The RAW readings are uncalibrated and have no associated unit which is why they cannot be used for determining the bias. Experiments prove that each sensor possesses a unique bias which can be used to generate the ICMetric of a device. Collecting 1500 readings from a sensor requires time which is why experiments were conducted with smaller data sets. Repeated experiments have shown that statistical stability is attained at 300 readings. Fig 2 shows the calibrated accelerometer and gyroscope readings obtained from two different devices for 1500 readings. The graphs show that each sensor possesses a different bias even when the axis is the same.

A statistical analysis of the unimodal distributions is necessary to prove that there is sufficient bias required to generate a unique ICMetric. Analysis shows that sensors from different devices will generate unique statistical credentials. All devices in the setup demonstrated that they possess a unique operational signature when subjected to a standard stimulus. Owing to limitation of space we provide only a small snapshot of the statistical analysis. Table 1 shows how the statistical credentials change even though the stimulus and sensor axis remains the same. The statistical measures are obtained by processing 1500 sensor readings per axis. The confidence interval and a p-value has been calculated using single factor Analysis of Variance (ANOVA). The obtained p-value is 0 which shows there is significant difference between two sets of readings.

**Table 1:** Statistical analysis of the normal distribution for two different devices embedded with an accelerometer and gyroscope.

		Device A	Device B
Accelerometer x-axis	Confidence Interval	0.003450	0.003769
	Standard Deviation	0.069307	0.075711
	Inter Quartile Range	0.089109	0.089109
	Mean	-0.291961	-0.746236
	Skewness	0.022999	2.325030
Gyroscope y-axis	Confidence Interval	0.866212	0.197496
	Standard Deviation	17.397349	3.966596
	Inter Quartile Range	22.34432	4.029304
	Mean	-75.569276	-77.463322
	Skewness	-0.079076	1.511550

## 5 Scheme design

**Fig 3:**
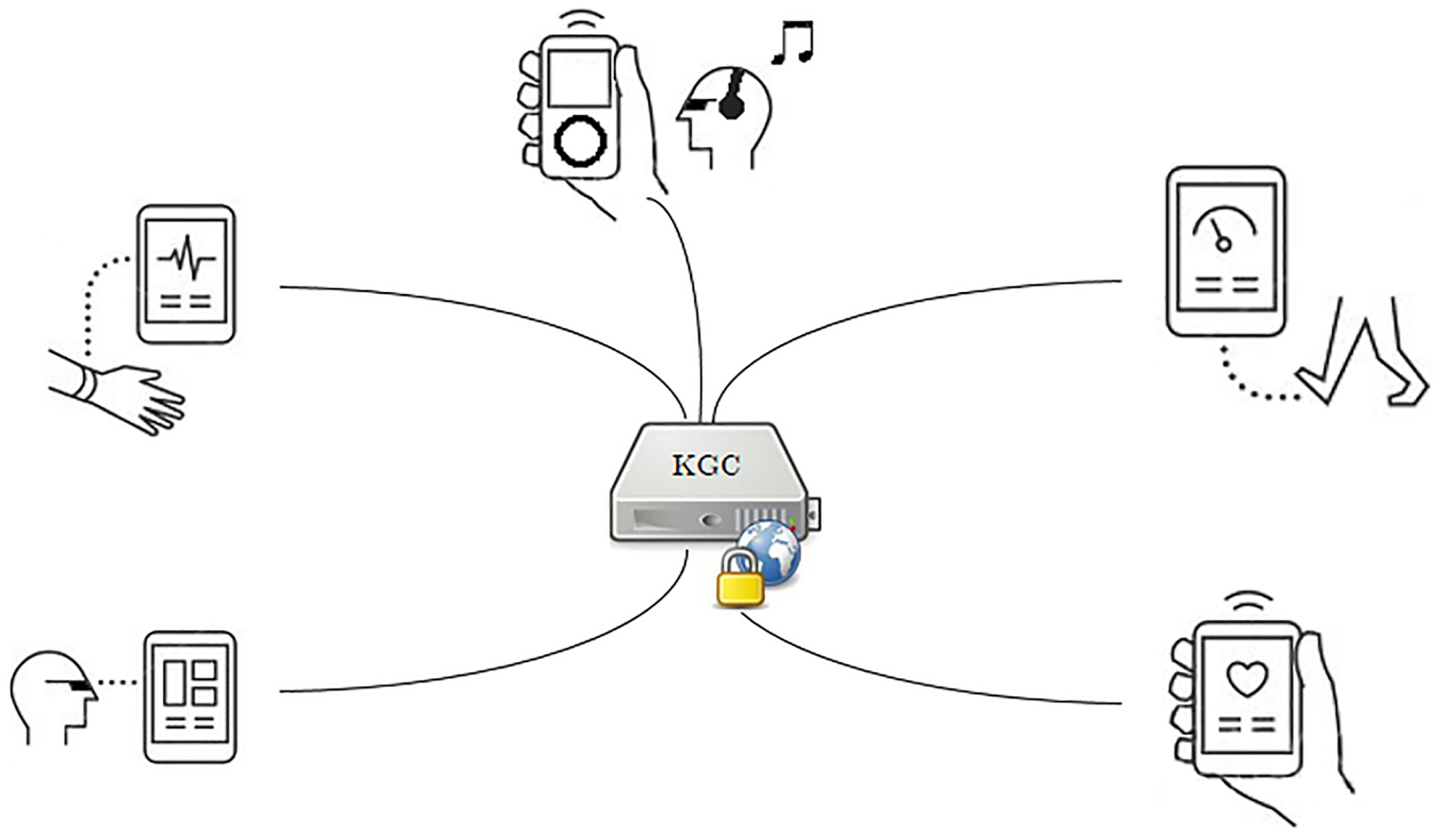
Basic system model for wearable devices communicating in the presence of a KGC.

Wearable devices in the IoT can be attacked in many ways. Many wearable devices do not possess adequate resources or even the ability to secure the user, data and the device. Some wearable devices are intended to provide health services in which case the importance of security is paramount. Attackers often use eavesdropping to intercept traffic which can then lead to a range of other attacks. The proposed scheme allows a number of wearable devices to communicate in a group environment. To control the group and its entities the scheme requires the installation of a Key Generation Center (KGC). All devices in the group are ICMetric capable and the group adheres to the properties of the ICMetric technology. While designing the scheme it is assumed that all communications happen through secure channels. Basing the communication on secure channels prevents eavesdropping of communications. Fig 3 shows the basic system model. The proposed scheme is designed to secure wearable devices in the group setting. The scheme is founded on the ICMetric technology and composed of five different phases i.e. device imprinting, device authentication, group ICMetric generation, symmetric key generation and confidentiality. Table 2 provides a description of the symbols and their meaning in the scheme. The following section provides details of the individual phases of the security scheme.

**Table 2:** A description of the symbols used in the scheme.

Symbol	Meaning
⊕	Bitwise exclusive OR
—	Concatenation
⌈ ⌉	Ceiling function
*ich*	Hashed ICMetric
*s*	128 bit random salt
*icm*_*d*_, *icm*_*KGC*_	ICMetric of device and KGC respectively
*ICM*_*g*_	ICMetric of the group
*ID*_*x*_	Identity associated with device *x*
*C*	Iteration count; minimum 1000
*kLen*	Length of master key in bits
*hlen*	Digest size of hash function
*be*(*x*)	32-bit encoding of integer *x*. Significant bit appears on left
*mk*	Master key

### 5.1 Device imprinting

The first step for establishing the group is imprinting [31]. The step is aimed at resolving the issue of establishing trust between two distrusting devices. The process mimics the duckling imprinting phase where a newborn duckling establishes a pattern with its parents. When a device wishes to join a group it will first need to register with the KGC. The KGC is responsible for coordinating and supporting the presence of the group and its individual members. When a device wishes to join the group, the device will compute a hash of its ICMetric *icm*_*d*_ and send it in plaintext to the KGC as follows:

$$\begin{array}{c} h=hash(icm_{d}) \end{array}$$ (2)

This value of *h* is discarded by the device but is stored by the KGC for future authentication.

### 5.2 Device authentication

To prove authenticity, the device will compute a hash of *h* and a plaintext temporary salt *s*_*temp*_ issued by the KGC. The device will respond by computing:

$$\begin{array}{c} h_{1}=hash\left(h+s_{temp}\right) \end{array}$$ (3)

The KGC will compute the same and compare the resulting value with that provided by the device. If both values are identical then the device will be authenticated. Upon successful authentication each device will be allocated a unique identity ID, which will help in establishing the group ICMetric.

### 5.3 Group ICMetric generation

A major goal of the proposed scheme is to form a group ICMetric. The purpose of forming a group ICMetric is to provide an identification to the established group. It is based on the ICMetric technology so that a group fingerprint can be created that is authentic and based on a physical root. The group identification could have been based on a device identification (serial numbers, MAC address, etc) but this has been avoided as these features can be spoofed. Generating the group ICMetric is a challenging task because this identification has to be based on the ICMetric of the constituent devices. The challenge lies in the fact that the ICMetric cannot be transmitted even to trusted entities. If the group ICMetric is generated without taking input from the individual group members then a group identity is generated that is not a true identification of the group and its constituent members.

To generate the group ICMetric without exposing the individual ICMetric we use Shamir’s Secret Sharing scheme. This scheme allows a number of individual members/ devices to construct the secret group ICMetric.

To generate the group ICMetric the devices in the group will be sent a temporary salt *s*_*temp*_ by the KGC. This will be used by the device to generate a hash by adding the *icm*_*d*_ and *s*_*temp*_ as follows:

$$\begin{array}{c} ich=hash(icm_{d}+s_{temp}) \end{array}$$ (4)

Thus the KGC will receive *ID* and *ich* pairs i.e. (*ID*_*x*_, *ich*_*x*_). The responses obtained from the individual devices will be used to form the secret share points required for constructing the group ICMetric. The responses obtained from the individual devices will be used to form the secret share points required for constructing the group ICMetric.

$$\begin{array}{c} \left(ID_{1},ich_{1}\right),\left(ID_{2},ich_{2}\right),\ldots ,\left(ID_{t},ich_{t}\right) \end{array}$$ (5)

The secret group ICMetric is constructed by using Lagrage interpolation. Lagrange polynomial is used with the previously assembled share points. The group ICMetric is assembled using the following polynomial:

$$\begin{array}{c} ICM_{g}=\prod_{j=1}^{t}ID_{j}\sum_{i=1,i\neq j}^{t}\frac{ich_{i}}{\left(ich_{j}-ich_{i}\right)\prod_{j=1,j\neq i}^{t}\left(ich_{i}-ich_{j}\right)} \end{array}$$ (6)

Where *t* is the number of individual pairs used for establishing the group ICMetric.

### 5.4 Password-Based Key Derivation Function

A password is an identifying phrase which can be composed of a combination of alphabets, numbers and special characters. User defined passwords do not possess enough entropy due to which they cannot be used as cryptographic keys. Password-Based Key Derivation Function (PBKDF) is a scheme that generates keys from secret values such as a password [32]. PBKDF uses a cryptographic hash or an HMAC with a salt to generate a key which is used for the provision of security services. Since PBKDF use a salt to generate a key it defeats rainbow table based attacks.

PBKDF is also based on an iteration count *C* which is intended to increase the amount of computation needed to derive the key. Increasing the computation makes brute force difficult but also effects the amount of computation required for the legitimate user. The aim while choosing the number of iterations is to increase the difficulty of attacks while ensuring low key generation impact. The NIST standard on PBKDF and other proposals recommend a minimum iteration count of 1000 iterations because this has minimum adverse effect on system performance. Higher number of iterations have been proposed in varying applications. A gradual increment in the iteration count has been recommended as and when computation power becomes available. When this is possible a maximum iteration count of 10,000,000 has been proposed but with the caution that the user-perceived performance is not of affected.

**Fig 4:**
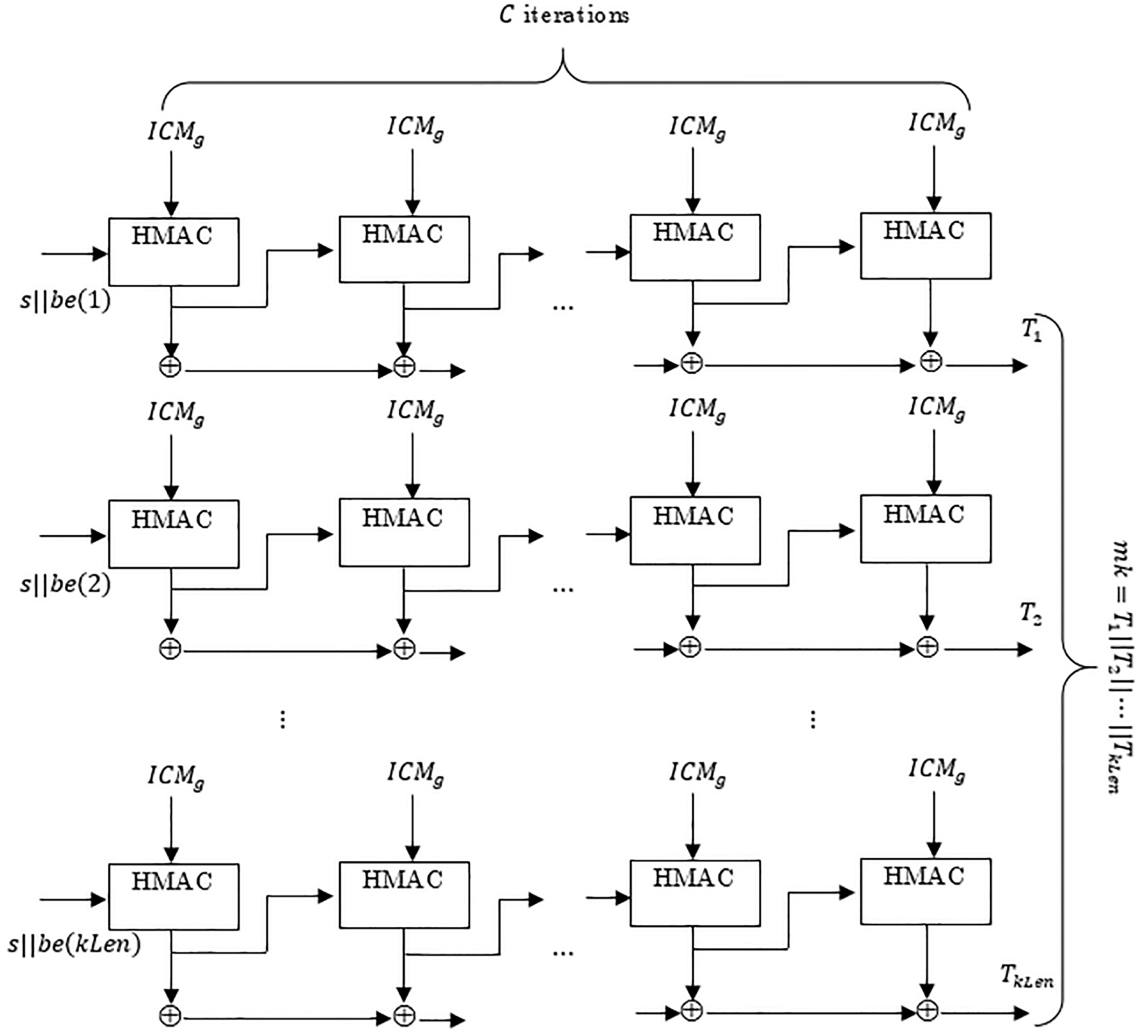
The PBKDF schematic showing the generation of a symmetric key using the group ICMetric.

The design considerations of a PBKDF bears close resemblance to the conditions of the ICMetric technology. For instance the password is a secret phrase which cannot be transmitted, similarly the ICMetric of a device cannot be transmitted. A quality of the PBKDF is that the large sized iteration count prevents an attacker from determining the password which in this case is the group ICMetric *ICM*_*g*_. Fig 4 shows the PBKDF schematic modified according to the ICMetric technology for the establishment of a symmetric master key. The proposed system is based on the NIST PBKDF [32] recommendation.

The proposed scheme uses *ICM*_*g*_ to generate a master key *mk* which is used for confidentiality services. *kLen* is the required length of the master key in bits. The *kLen* can be modified to conform with the needs of the target encryption algorithm. The proposed algorithm also takes as input the digest length *hLen*. A modifiable key length and hash digest length allows adaptability of the algorithm to upcoming and changing cryptographic requirements. The algorithm for symmetric key generation using PBKDF is as follows.

if (kLen>2^32^−1)x h

  Return with Error

len = kLen/hLen

r = kLen−(len−1)xhLen

for (i = 1 to kLen)

{

  T_i_ = 0

  U_0_ = s || be(i)

  for (j = 1 to C)

   {

   U_j_ = HMAC(ICM_g_, U_j−1_)

   T_i_ = T_i_⊕U_j_

   }

}

Return mk = (T_1_||T_2_||…|| T_kLen_)

Since all involved parties and the KGC possess the same group ICMetric therefore it can be concluded that using the group ICMetric will result in a single symmetric key for all parties in the group.

### 5.5 IoT encryption/ decryption

After generating the ICMetric based symmetric key the next step is to use the key for the provision of confidentiality services. Wearable devices in the IoT ecosystem often generate data in the form of a data stream. To test the confidentiality services of the system we use the AES and Rabbit Stream Cipher [33] [34]. The Rabbit stream cipher is a recognized eSTREAM cipher with high throughput. The purpose of the eSTREAM project was to identify promising stream ciphers which can be widely adopted. The scheme has also been tested with 128 bit and 256 bit AES variants. The aim while choosing encryption schemes was to select schemes that offer high levels of confidentiality without the resource demand.

## 6 Implementation and outcomes

The system has been implemented and tested on Intel Core i5 3.4 Ghz processor computer with 6GB RAM. The MEMS readings were obtained from the Shimmer sensor while authentication, group ICMetric generation, key generation and confidentiality scheme has been implemented in Bloodshed Dev-C and MATLAB. The cryptographic functionalities were provided by the OpenSSL cryptographic library.

The system has been divided into five subcategories each targeting a different cryptographic service. The system is composed of the following modules:

- ICMetric generation—A module dedicated to generating an ICMetric from the sensor readings.
- Authentication—A module designed to authenticate the individual devices in the group environment.
- Group ICMetric Generation—A module dedicated to generating a group ICMetric using the Shamir Secret Sharing Scheme.
- Key Generation—A PBKDF scheme for the generation of varying conventional key sizes i.e. 128, 256, 512, 1024 bits.
- Confidentiality—Two stream cipher modules i.e. Rabbit stream cipher and AES (128 and 256 bit).

### 6.1 Results

**Fig 5:**
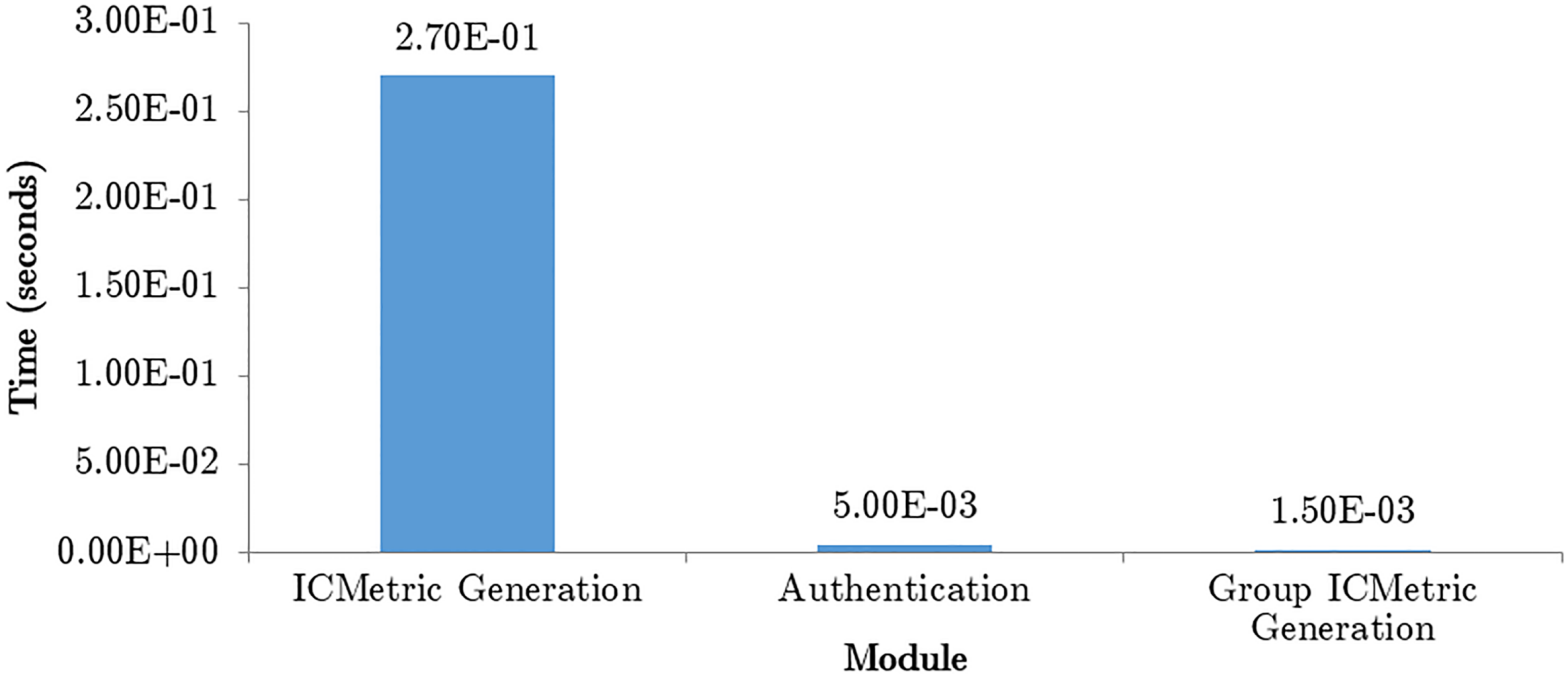
Time taken by the system modules.

The sampling rate for the accelerometer and gyroscope in the Shimmer is 51.2Hz. Once the readings are obtained from the sensor in a CSV file the readings are used to generate the final ICMetric. Implementation of the algorithm takes 0.27512 seconds to generate an ICMetric using both the accelerometer and the gyroscope. Once the ICMetric has been generated the device will use authentication services to get itself authenticated using the ICMetric. The authentication scheme contains two occurrences of the SHA256 function. The authentication module takes 5 × 10^−3^ seconds to execute. The Group ICMetric generation is based on Shamir Secret Sharing Scheme. This module requires 1.5 × 10^−3^ seconds to execute completely. Fig 5 shows the time required by the individual modules of the scheme.

The PBKDF is influenced by two parameters i.e the key size and the number of iterations. To study the performance of this algorithm four common key sizes were generated using varying number of iterations. The generated key sizes are 128, 256, 512, 1024 bits while the tested iteration count is 1000, 2000 and 4000. Table 3 shows the time taken by the PBKDF when subjected to varying key sizes and iteration count.

**Table 3:** Time taken by PBKDF with varying key size and iteration count.

Iteration Count	Key Size (bits)	Key Generation Time (seconds)
1000	128	0.019
	256	0.040
	512	0.081
	1024	0.160
2000	128	0.041
	256	0.080
	512	0.146
	1024	0.310
4000	128	0.093
	256	0.166
	512	0.328
	1024	0.588

**Fig 6:**
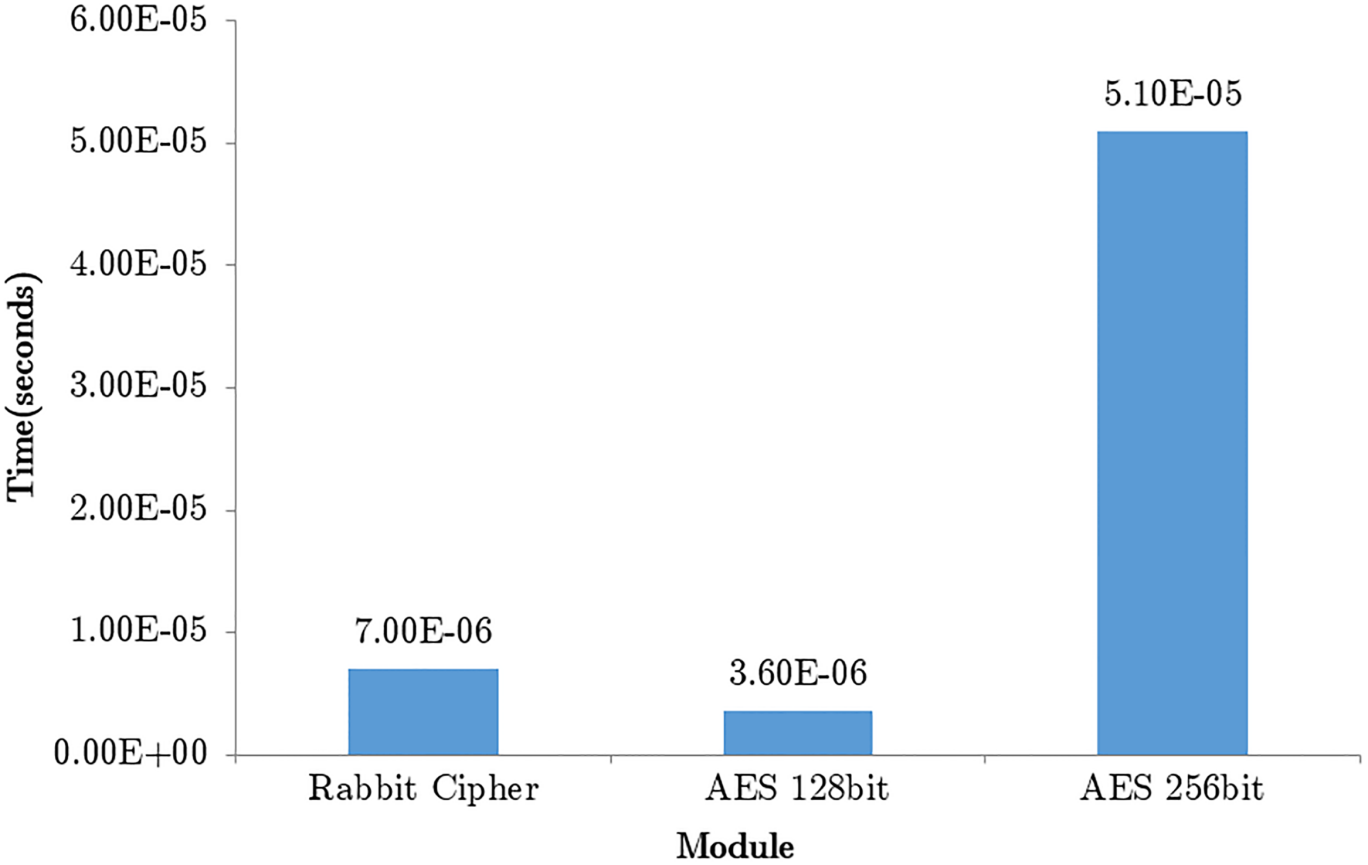
Graph showing time (seconds) taken by Rabbit and two variants of AES.

A time study of the keys generation module shows that the 256-bit key with 4000 iterations requires the most time to operate. Increasing the number of iterations and the key size impacts the time required by the system. Fig 6 shows a change in time required as the key size and the number of iterations increases.

**Fig 7:**
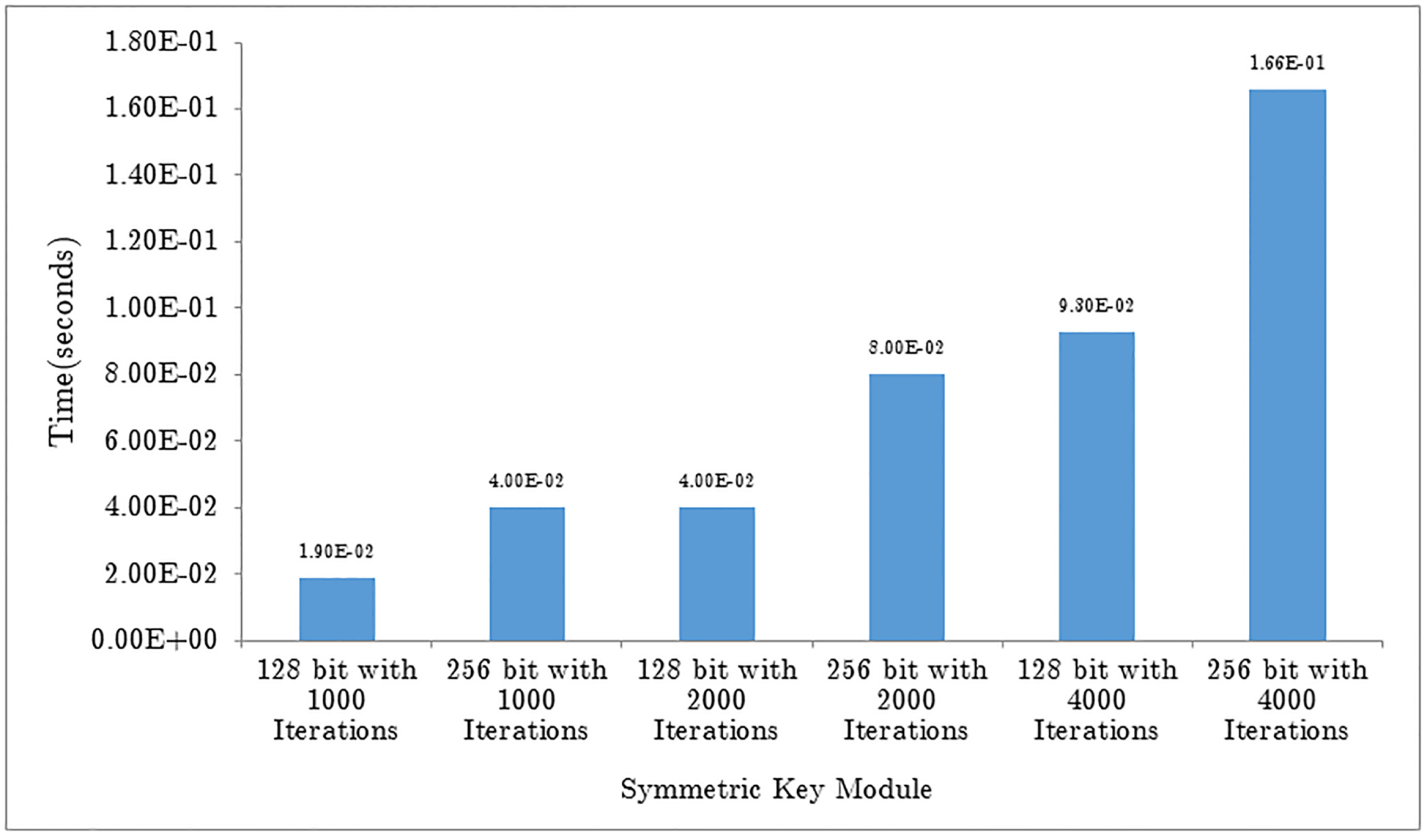
Graph showing time (seconds) taken to generate various key variants.

The confidentiality module has been implemented with two widely recognized encryption algorithms i.e. Rabbit stream cipher and AES. The AES encryption module is composed of two variants i.e. 128 bit and 256 bit. The 128 bit variant requires 3.6 × 10^−6^ seconds to run; while the 256 bit variant requires 51 × 10^−6^ seconds. The rabbit stream cipher requires a 128 bit key and a 64 bit initialization vector to run and requires only 7 × 10^−6^ seconds to run top down. Given in Fig 7 is a graph showing the time taken by the individual encryption schemes and their variants.

### 6.2 Discussion

The security scheme unifies the ICMetric and prominent cryptographic schemes like Shamir Secret Sharing, PBKDF, AES and Rabbit to create a system that ensures security for wearable devices in the IoT. The ICMetric technology deters key theft by using the features of a sensor. The device ICMetric is generated only when required and discarded thereafter. Readings from sensors are used to determine the bias and thus generate an identification for the device. The ICMetric technology is a key theft deterrence technology and also provides device authentication services. The proposed scheme provides authentication by using the device ICMetric. When the authentication happens in a group setting we have an environment where only authenticated devices can communicate and share resources.

Since the designed scheme targets devices in a group setting therefore we propose generating an identification for the devices that form the group. To overcome the problems associated with exposure of the individual ICMetric, the scheme uses Shamir Secret Sharing to generate an ICMetric for the group. This scheme allows the creation of a group ICMetric which is an identification for the group. The presented system uses the group ICMetric to generate a symmetric key using PBKDF. This module supports attack deterrence through an adaptable iteration count. The scheme also promotes effortless adaptability to varying key sizes. By incorporating cryptographic salts throughout the designed system it deters dictionary based brute force attacks.

The ICMetric of a device is never communicated thus discouraging man in the middle attack. To further strengthen the scheme noteworthy cryptographic elements have been incorporated like random salts and ephermal keys. Using these architectural elements ensures protection against replay and impersonation attacks. Provision of strong authentication alone does not ensure a fully secure system therefore it has been studied in combination with prominent confidentiality schemes like AES and Rabbit.

For the correct functioning of the proposed schemes it is essential that the same ICMetric is generated time and time again. It is a well-known fact that functioning of MEMS sensors is affected by a number of factors like aging, environmental impact, heat, electromagnetic radiations and physical damage. These factors will influence the functioning of the device and ultimately the production of the ICMetric. Methods for compensating the sensor drift could be incorporated to ensure the correct generation of an ICMetric. Here one may be tempted to base the device identification on a simpler feature like a device identification. This in our opinion is not recommended as identifications are easily obtained (often printed on the exterior of a device).

### 6.3 Result comparison

Studying the PBKDF algorithm with the ICMetric technology is a novel concept that has not been explored previously. The simulation results of the proposed symmetric key scheme is compared to a healthcare sensing system based on the ICMetric technology [15]. The system is a one to one scheme that provides ICMetric based authentication and access control. The system also offers AES based encryption by using symmetric keys. Since the system was intended for one to one communication therefore the scheme is not constituent of a group ICMetric module. The scheme is initiated with the establishment of an ICMetric, followed by generation of the symmetric key. This symmetric key is then used to provide confidentiality services. The authors have simulated the proposed scheme and the projected time consumption is similar to that of our scheme. Authentication services and key exchange is carried out by a Secure Remote Password scheme and hence the authors have not provided a dedicated module for authentication. Table 4 provides the time taken by this scheme and the rivalling scheme also based on ICMetric technology. As the contending scheme does not simulate all modules therefore absent details have been represented with a dash.

**Table 4:** A running time (seconds) comparison of the proposed symmetric key scheme with an ICMetric based one to one healthcare system.

	Proposed scheme			ICMetric based one-to-one scheme		
Group ICMetric generation	1.5 × 10^−3^ sec			-		
Authentication	5.0 × 10^−3^ sec			-		
Symmetric key generation	1000 iterations			160 bit	256 bit	512 bit
	128 bit	256 bit	512 bit			
	1.9 × 10^−2^ sec	4.0 × 10^−2^ sec	8.1 × 10^−2^ sec	2.65 × 10^−3^ sec	3.6 × 10^−3^ sec	3.85 × 10^−3^ sec
AES 128	3.6 × 10^−6^ sec			3.1 × 10^−6^ sec		
AES 256	5.10 × 10^−5^ sec			-		
Rabbit Encryption	7.0 × 10^−6^ sec			-		

## 7 Conclusion

Wearable devices with internet connectivity is a recent advancement in the field of computing. Wearable devices can have many purposes ranging from health monitoring to entertainment. Where wearable devices offer much advantage, a hurdle in their wide adoption are security concerns. This paper presents the design and implementation of a security scheme for multiple wearable devices in the IoT. Cryptographic algorithms are often based on secret keys. If the keys of a system are captured then this can result in the system being exposed. The ICMetric technology is a novel concept that eliminates the need for stored keys. By using the ICMetric technology keys are generated when required and discarded thereafter. This paper studies the design and implementation of an ICMetric based system that uses the features of a device to create secret group keys which are then used for cryptographic services. The accelerometer and gyroscope bias in sensors has been used to determine the ICMetric identification of a device. Once the device ICMetric is generated, Shamir Secret Sharing scheme has been used to generate an ICMetric for the group. This group identification is used to generate a symmetric key for the group using PBKDF. In the paper experiments have been conducted to determine the relationship between the key size and the number of iterations required for the generation of a symmetric key. Confidentiality services of the designed system are based on the AES 128, 256 and the Rabbit stream cipher. The scheme is based by integrating strong cryptographic elements so that the scheme provides the highest levels of security with a limited resource demand. The designed scheme function without human intervention thus the ICMetric and associated system components operate without the need for user input. Using this algorithm has the added advantage that the group ICMetric can be generated with varying number of group members present. Thus to assemble the group ICMetric a predetermined set of group members do not have to be present. The proposed scheme can be adapted to other challenging environments where there are many systems in a multiparty environment. Simulation results of the proposed schemes shows that the ICMetric technology can be used to provide security to devices that communicate in a group setting. The ICMetric technology deters key theft and forms a basis for cryptographic services but does not add to the resource demand of the target system.

## Supporting information

S1 File Simulation data is available in spreadsheet file titled S1_File.xlsx. (XLSX)
